# Associations between demographic factors and the academic trajectories of medical students in Japan

**DOI:** 10.1371/journal.pone.0233371

**Published:** 2020-05-18

**Authors:** Nobutoshi Nawa, Mitsuyuki Numasawa, Mina Nakagawa, Masayo Sunaga, Takeo Fujiwara, Yujiro Tanaka, Atsuhiro Kinoshita

**Affiliations:** 1 Curricular Institutional Research Division, Institute of Education, Tokyo Medical and Dental University, Tokyo, Japan; 2 Department of Medical Education Research and Development, Tokyo Medical and Dental University, Tokyo, Japan; 3 Department of Global Health Promotion, Tokyo Medical and Dental University, Tokyo, Japan; University of Westminster, UNITED KINGDOM

## Abstract

**Background:**

Group-based trajectory modeling is a useful tool for categorizing students’ academic trajectories and their determinants. Using insights gained from the analyses, we can identify students at risk for poor academic performance and monitor them to provide support. To date, studies investigating the associations between demographic factors and academic performance trajectories among medical students are scarce. The study objective was to examine the associations between demographic factors and academic performance trajectories in medical students using group-based trajectory modeling.

**Methods:**

Participants included all medical students admitted to Tokyo Medical and Dental University in Japan in 2013 and 2014 (n = 202). Academic performance was evaluated by biannual grade point average (GPA) scores in preclinical years. We used group-based trajectory modeling to categorize students into GPA trajectories. Multinomial logistic regression was used to examine the association between the odds of being in a certain GPA trajectory group and demographic factors such as high school type, high school geographical area, admission test type, high school graduation year, whether the student was a biology major, and sex.

**Results:**

Students’ GPA trajectories were classified into four trajectory groups as well as another group that consisted of students who withdrew or repeated years. We found that students whose high school geographical area was outside the National Capital Region were 7.2 times more likely to withdraw or repeat years in comparison with students whose school was inside the National Capital Region (OR: 7.21, 95% CI: 1.87, 27.76). In addition, admission test type, high school graduation year, and sex were associated with GPA trajectories.

**Conclusions:**

High school geographical area, admission test type, high school graduation year, and sex were associated with GPA trajectories. These findings provide important insights into identifying students at risk for poor academic performance and strategies for monitoring them to provide adequate and timely support.

## Introduction

With advancements in medicine, the study load for medical students has been increasing. In Japan, the decline in academic performance and increase of the number of medical students who repeat years and withdraw from medical school have been important issues [1, 2]. To maintain the quality of healthcare provided by the students post-graduation, ensuring their success by monitoring their academic performance and providing them with timely support (e.g., academic, social and psychological support) is important.

A prior systematic review reported several factors that could affect medical students’ academic success, including previous academic performance, and cognitive ability [3]. Studies have also reported associations between demographic factors, such as gender and age, and students’ academic performance and/or dropout rate [4–10]. Furthermore, contextual factors such as type of school (e.g., state-funded schools vs. independent schools) [11] and hometown (rural or remote vs. metropolitan areas) [4, 12] have been linked to student academic performance. Studies investigating determinants of students’ academic performance are important since they allow us to identify students at risk for poor academic performance and monitor them in order to provide adequate support in a timely manner.

Medical students in Japan usually attend lectures in a classroom in the first four years. In the first year in our university, medical students learn various subjects including mathematics, physics, chemistry, liberal arts, and foreign languages. From the second year, medical students are required to attend lectures for medical subjects.

In this study, we formulated our conceptual framework from the literature on student adjustment to university [13–16]. According to the theory, students need to cope with the changes to their life, such as the increasing study load, by making adjustments in multiple domains including academic, social, and personal-emotional domains, as well as attachment to the institution that they attend [13–16]. Prior research suggests that intellectual ability as well as background demographic factors can affect their adjustment [17]. Thus, students may follow different academic trajectories depending on their characteristics.

Group-based trajectory modeling [18, 19] allows researchers to categorize each individual into a different group with a different outcome trajectory, and to clarify factors that determine the probability of an individual following one of these trajectories [20, 21]. Pioneering research by Griffin *et al*. used a similar trajectory analysis to assess whether students’ selection test scores could predict their academic performance trajectories [22].

However, studies applying group-based trajectory modeling to examine the associations between demographic factors and academic performance trajectories are lacking. These insights are important since they would enable us to identify students at risk for poor academic performance and monitor their academic trajectories to provide adequate and timely support. Earlier identification of these students is also crucial as prior studies have reported that students with poor academic performance tend not to seek advice and/or support [10, 23]. These studies used data from routine assessment of students at earlier stages rather than data obtained by additional research tools to predict their later performance [10, 24]. Prediction of students’ later academic performance using data, such as that collected for the admission process, is useful because these approaches could also be applied in resource-poor settings. Furthermore, data collected for the admission process, such as demographic factors (age, sex) as well as school factors, often include important information that has been linked to students’ academic performance [3–12]. Thus, we hypothesized that demographic factors can be used to predict trajectories of students’ academic performance. The objective of this study was to investigate the associations between demographic factors and the academic performance trajectories of medical students in Japan.

## Method

### Sample

Participants consisted of 202 medical students who were admitted to Tokyo Medical and Dental University (TMDU), Tokyo, Japan in 2013 and 2014. Since 2011, the current curriculum has been implemented and students in 2013 and 2014 basically took the same courses. Twenty-one medical students withdrew from the university or repeated school years. Following approval from the ethics committee at TMDU, students were informed of their right to refuse to allow researchers to conduct a secondary analysis of their data, using an online bulletin board for students, and the school website. De-identified data were provided to researchers by the Curricular Institutional Research Division of the Institute of Education at TMDU after removing students who wanted to opt-out. This study was approved by the ethics committee at TMDU (M2018-279). There were no missing values in the variables used in this study.

### Measures

#### Independent variables

*Demographic factors*. Demographic factors included the type of high school (public, private, or national), geographical area of high school (‘inside the National Capital Region’, defined as being located in Tokyo, Ibaraki, Tochigi, Gunma, Saitama, Chiba, Kanagawa, or Yamanashi prefecture vs. ‘outside of the National Capital Region’), type of admission test (first exam, second exam, or admission via specific prefectural quotas [i.e., Students who enter by these quotas are selected by different criteria and are encouraged to work for the prefecture after graduation]), high school graduation year (recent graduates [i.e., high school graduation year is the same as the year of admission to university] or past graduates [i.e., high school graduation year is at least one year before the year of admission to university]), biology major (yes or no), and sex. In Japan, to apply for medical school, the majority of students take the standardized national test and additional exams for each university [25]. Students can take the national center exam once each year. In contrast, students generally can take the university’s specific exams twice each year [26]. As students have two chances to apply for university each year, some students apply to the same university twice to increase their chance of being admitted, while others apply to a different university each time [27]. As the second exam is more competitive, some students apply to a university that is ranked below the university to which they first applied [27]. Universities evaluate students for their eligibility based on the score of these two tests.

*Past academic performance*. Past academic performance was assessed using high school GPA scores that were reported from their high schools during the admission application process.

#### Dependent variables

*Academic performance*.: Academic performance was evaluated by a biannual GPA for each semester. We used biannual GPA instead of cumulative GPA, because biannual GPA is more sensitive to subtle changes in the students’ academic performance, which is more useful for detecting students who may need support. In Japan, medical students study for six years in university: the first year is the premedical phase, the second to fourth is the preclinical phase, and the fifth to sixth is the clinical clerkship phase. Grade points for classes ranged from 0.0 to 4.0. By averaging grade points for courses in each semester, GPA was calculated, which also ranged from 0.0 to 4.0. If a grade point score for a required course was less than 2.0, students needed to repeat a year and receive a grade point greater than or equal to 2.0 to pass. In this study, we focused on GPA in the preclinical phase (second to fourth years: six scores) since no GPA were provided in the fifth year and the GPA data for the sixth year was not available at the time of the study (both were in the clinical phase). In addition, in the premedical phase (from the first to the second semester), the class composition was different (the medical and dental students took the same classes, and grade points for classes were provided, taking the composition of the class into account).

### Statistical analyses

To categorize students into different groups with different GPA trajectories, group-based trajectory modeling was used [18–21, 28]. First, we estimated the number of trajectory groups and the shape of the trajectory by maximizing the Bayesian information criterion (BIC) [28]. We increased the number of trajectory groups from two to six. All the trajectories had a quadratic shape, and the number of trajectory groups was chosen to maximize the BIC. After determining the number of trajectories, we assessed whether changing the shape of each trajectory to a linear one increased BIC. Then, covariates were added, to establish whether their addition increased BIC. The GPAs of the 181 students who did not withdraw or repeat years were analysed by group-based trajectory modelling. The 21 students who withdrew or repeated years were categorized into Group 4 (Withdrew or repeated). After determining the number of groups and shape of the trajectory, multinomial logistic regression was used to examine the association between the odds of being assigned to certain categories and demographic factors.

During the eighth semester, all students were assigned to one of the laboratories in our university or other universities to conduct research work and a grade was provided at each laboratory. Thus, the grading was not standardized. For sensitivity analysis, we also conducted trajectory analyses using GPA from the third semester to the seventh semester instead of from the third semester to the eighth semester. All statistical analyses were conducted using Stata SE statistical package, version 14 (StataCorp LP, College Station, TX, USA), and the stata “traj” plugin for group-based trajectory modeling [20].

## Results

Table 1 shows the characteristics of the sample population. Among 202 students, 18% graduated from public high schools, and about 20% graduated from high schools that were located outside of the National Capital Region. About 81% took the first exam as an admission test. The percentage of the recent graduates was 65.4%. About 19% majored biology in their high school. 71.8% were male.

**Table 1:** Characteristics of the sample population.

Variable	Medical students (n = 202)
	Mean (SD) or N (%)
Type of high school	
Public	37 (18.3)
Private	144 (71.3)
National	21 (10.4)
Geographical area of high school	
Inside the National Capital Region (Tokyo, Ibaraki, Tochigi, Gunma, Saitama, Chiba, Kanagawa, or Yamanashi)	162 (80.2)
Outside the region	40 (19.8)
Type of admission test	
First exam	164 (81.2)
Second exam	30 (14.9)
Quota for certain prefectures	8 (4.0)
High school graduation year	
Recent graduates (The same year)	132 (65.4)
Past graduates (≥ one year(s) ago)	70 (34.7)
Biology major	
Yes	39 (19.3)
No	163 (80.7)
Sex	
Male	145 (71.8)
Female	57 (28.2)
High school GPA	4.6 (0.4)
Semester GPA	
1st	3.3 (0.3)
2nd	3.3 (0.3)
3rd	2.9 (0.4)
4th	3.1 (0.3)
5th	3.2 (0.4)
6th	3.2 (0.3)
7th	3.3 (0.4)
8th	3.6 (0.2)

**Fig 1:**
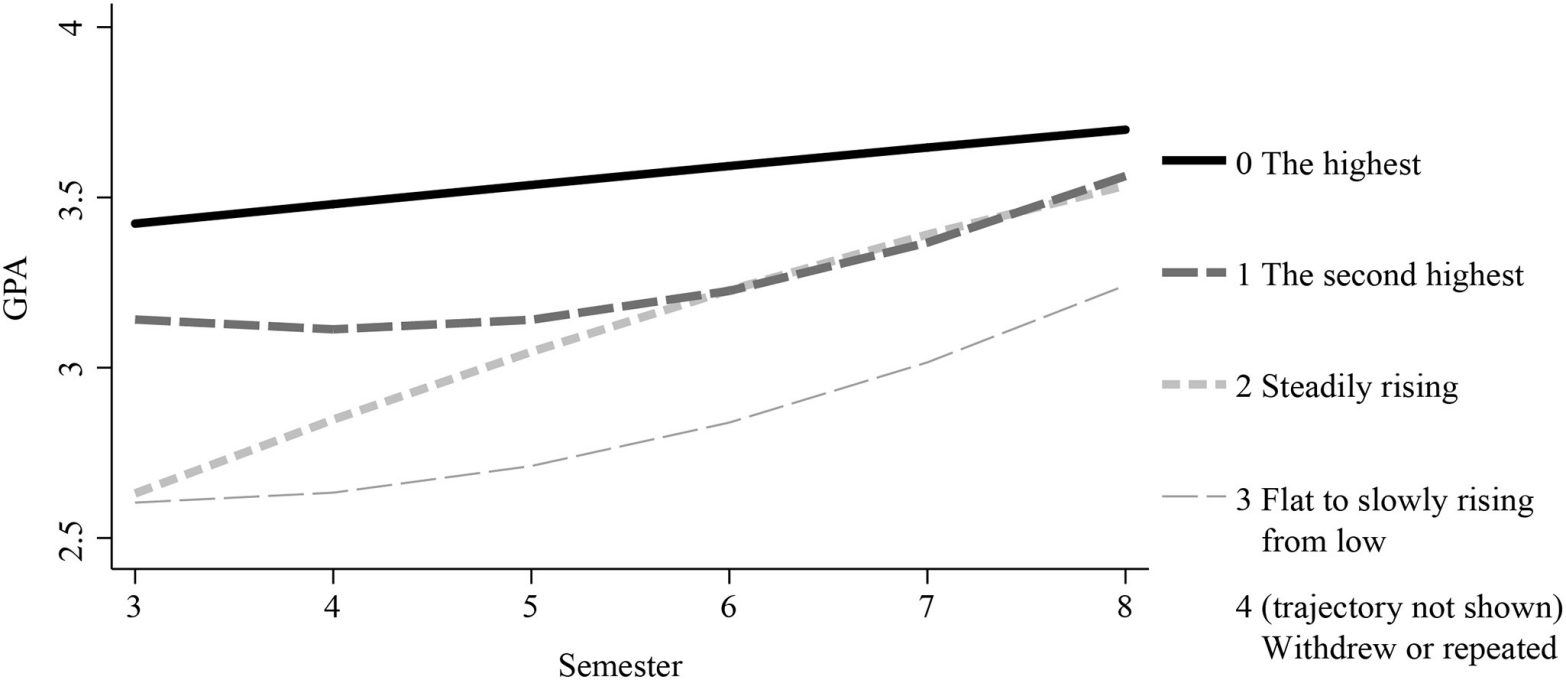
The GPA trajectories of medical students that were modeled using GPA data from 3^rd^ semester to 8^th^ semester.

Fig 1 shows the GPA trajectories of medical students from the third semester to the eighth semester (from second year to fourth year, the preclinical phase). We estimated the number of trajectory groups and the shape of the trajectory by maximizing the Bayesian information criterion (BIC) [28]. Based on the largest BIC, we chose four as the number of trajectories. Then, the shape of the trajectories (quadratic, except for Group 0, which was linear) and inclusion of high school GPA as a covariate in the model were determined, based on which resulted in the largest BIC.

After categorizing students into different groups with different GPA trajectories using group-based trajectory modeling, multinomial logistic regression was used to examine the association between the odds of being assigned to a certain GPA group and demographic factors.

In Table 2 and S1 Table, the odds of being a member of a certain GPA trajectory group relative to the highest scoring reference GPA group are shown in relation to demographic factors, with and without adjustment for high school GPA. In analyses without adjustment for high school GPA (S1 Table), although there was no significant association between being a member of a certain group and the type of high school, when the geographical area of the high school was outside the National Capital Region, students were 5.08 times more likely to withdrew or repeat years in comparison with students whose high school was inside the National Capital Region (OR: 5.08, 95% CI: 1.41, 18.24). When the geographical area was outside the National Capital Region, students were more likely to be among a group of students whose GPA was flat to slowly rising from low, although not reaching statistical significance (OR: 2.96, 95% CI: 0.89, 9.80). A similar result was observed in analyses with adjustment for high school GPA (Table 2). The association between the geographical area of the high school being outside the National Capital Region and being in a group of students who withdrew or repeated years (OR: 7.21, 95% CI: 1.87, 27.76), or whose GPA was flat to slowly rising from low (OR: 4.15, 95% CI: 1.17, 14.73) was significant after adjustment for high school GPA.

**Table 2:** The odds ratios of being a member of certain group of GPA trajectory relative to a reference group by demographic factors in medical students (N = 202) with adjustments for high school GPA (ref: The highest GPA trajectory group N = 39 (19.3%)). **(The GPA trajectories of medical students were modeled using GPA data from 3rd semester to 8th semester)**.

Variable	Group 1: The second highest	Group 2: Steadily rising	Group 3: Flat to slowly rising from low	Group 4: Withdrew or repeated
	(N = 67 (33.2%))	(N = 42 (20.8%))	(N = 33 (16.3%))	(N = 21 (10.4%))
	OR (95% CI)	OR (95% CI)	OR (95% CI)	OR (95% CI)
Type of high school (ref: Public)				
Private	1.32 (0.48, 3.64)	1.89 (0.56, 6.44)	2.35 (0.61, 9.07)	1.20 (0.28, 5.07)
National	0.24 (0.04, 1.39)	0.70 (0.11, 4.36)	0.63 (0.09, 4.27)	1.12 (0.17, 7.31)
Geographical area of high school (ref: Inside the National Capital Region)				
Outside the region	1.49 (0.47, 4.70)	1.05 (0.27, 4.06)	**4.15 (1.17, 14.73)**	**7.21 (1.87, 27.76)**
Type of admission test (ref: First exam)				
Second exam	**0.31 (0.11, 0.91)**	0.42 (0.12, 1.42)	0.69 (0.20, 2.34)	0.15 (0.02, 1.28)
Quota for Certain prefectures	0.23 (0.02, 2.71)	2.00 (0.32, 12.46)	0.72 (0.06, 8.99)	NA^a^
High school graduation year (ref: Recent graduates)				
Past graduates	1.92 (0.68, 5.41)	**4.48 (1.49, 13.47)**	**5.28 (1.65, 16.93)**	2.11 (0.56, 7.93)
Biology major (ref: No)				
Yes	0.59 (0.22, 1.59)	0.72 (0.24, 2.22)	0.64 (0.18, 2.24)	1.51 (0.43, 5.29)
Sex (ref: Female)				
Male	1.28 (0.55, 3.01)	2.30 (0.83, 6.38)	3.15 (1.00, 9.90)	**4.90 (1.15, 20.86)**

Adjusted for year of admission and high school GPA.

Bolded values indicate statistical significance at p<0.05.

^a^NA: not available because of the small sample size.

Entering the university by second exam was negatively associated with being in a group of students who withdrew or repeated years, or had lower trajectories than the reference highest GPA trajectory in the analyses with and without high school GPA adjustment (Table 2 and S1 Table). After adjustment for high school GPA, the association between entering the university by second exam and being in a group of students who had the second highest GPA trajectory was significant (OR: 0.31, 95% CI: 0.11, 0.91). There was a negative association between entering the university by second exam and being in a group of students who withdrew or repeated (OR: 0.15, 95% CI: 0.02, 1.28).

In analyses with and without adjustment for high school GPA, there was a positive association between being a past graduate and being in a group of students who withdrew or repeated years, or had lower trajectories compared to the reference highest GPA trajectory (Table 2 and S1 Table). The associations between being a past graduate and being in a group of students whose GPA was steadily rising (Group 2), and flat to slowly rising from low (Group 3) were significant in analyses with and without adjustment for high school GPA (With adjustment for high school GPA, for Group 2, OR: 4.48, 95% CI: 1.49, 13.47; for Group 3, OR: 5.28, 95% CI: 1.65, 16.93). There was no significant association between taking biology and students’ academic trajectory in the analyses both with and without adjustment for high school GPA.

Males were positively associated with being in a group of students who withdrew or repeated years or had lower trajectories compared to the reference highest GPA trajectory in analyses both with and without adjustment for high school GPA (Table 2 and S1 Table). In the analysis with adjustment for high school GPA, males were significantly associated with being in a group of students who withdrew or repeated years (OR: 4.90, 95% CI: 1.15, 20.86).

Finally, a one point decline in high school GPA was positively associated with the odds of being in a group of students who withdrew or repeated years, or who had lower trajectories than the reference highest GPA trajectory (S1 Table).

We also conducted sensitivity analysis by excluding the GPA for the eighth semester since during the eighth semester all students conducted research at different research laboratories and a grade was provided by each laboratory, meaning the grading was not standardized. The analysis showed similar results as shown in S1 Fig, S2 and S3 Tables.

## Discussion

In this study, we assessed how students’ academic performance trajectories were affected by demographic factors. We found that high school geographical area, type of admission test, high school graduation year, and sex were significantly associated with academic performance. In addition, high school GPA was significantly associated with students’ academic performance, which is consistent with prior studies [3, 29]. Medical students may experience different adjustment processes and follow different trajectories depending on demographic factors [13–17, 22]. Thus, our findings provide useful information for identifying students at risk for poor academic performance and monitoring them to provide support in a timely manner.

High school geographical area being outside of the National Capital Region was a risk factor for a lower GPA trajectory, withdrawing, or repeating school years. Since our university is in Tokyo, which is inside the National Capital Region, students whose high school was outside the National Capital Region (thus, their home may also have been outside the National Capital Region) may have needed to move to a location closer to university and started to live by themselves. Thus, one possible mechanism explaining the association may be isolation. However, previous studies in Saudi Arabia reported that students’ academic performance was not affected by whether students live with their family [30, 31]. Another possibility may be the socioeconomic disadvantage that is associated with living outside the National Capital Region. Previous studies in Australia found an association between the socioeconomic disadvantage associated with living in non-metropolitan/rural area and poor student academic performance [32, 33]. However, it should be noted that these studies were conducted with different sample populations. More studies examining the association between high school geographical area and students’ academic performance, taking into account differences in students’ mental health, diet, sleep patterns, and socioeconomic status are warranted.

Students who entered university by second exam were more likely to be in the highest GPA group. In addition, students who entered the university by second exam were less likely to be in a group of students who withdrew or repeated. In the university entrance exam system in Japan, students have two chances (i.e., the first exam and the second exam) to apply for university [26]. Some students apply to a university that is ranked below the university they wanted to attend for their second chance, because at the time of the second exam, places are limited and admission is more competitive. Thus, a possible difference in population characteristics due to the type of admission test taken may explain this finding.

In our analyses, there was a positive association between being a past graduate and being in a group of students who withdrew, repeated years, or had lower trajectories. In Japan, if students are not accepted by universities, they often go to a private ‘cram’ school to study for another year and retake the university entrance exams the following year. These students are called “*Ronin*” [34, 35]. Previous research found that “*Ronin*” students were sleep deprived, relatively depressed, and had various somatic complaints during the period before they retake the tests and successfully pass the exam [35, 36]. To examine the possible mechanisms of the association between high school graduation year and the students’ academic performance, studies assessing mental health and sleep problems among medical students who were admitted after being a “*Ronin*” student are needed in order to gain insights for appropriate support.

Finally, we found that with adjustment for high school GPA, males were significantly associated with being in a group of students who withdrew or repeated years. This is consistent with previous studies [3, 31]. One proposed reason explaining the higher performance of female students was motivation [3], and according to the theory on student adjustment, motivation was also crucial aspect of academic adjustment [13, 16]. Future studies assessing the association between student academic adjustment and the female students’ academic performance are warranted.

Our study has several limitations. First, our sample was derived from a single institution that has a competitive admission process and is located in Tokyo.

Thus, this may limit the generalizability of the findings. Second, our sample consists of two cohorts who were admitted to our university in 2013 and 2014. Although students basically took the same courses based on the same curriculum, and we included year of admission as a covariate in our model to take into account the possible difference between these two cohorts, there could be some residual confounding. Third, in our dataset we did not include academic performance in the final clinical years or reasons for the students’ withdrawal, both of which should be examined in future research.

Our study has several practical implications. Regarding educational implications, in Japan, an important issue in medical education has been decline in academic performance, and increase of the number of medical students who repeat years and withdraw from medical school [1, 2]. Given finite educational resources, if we could identify students who may follow a poor academic performance trajectory, we could monitor their academic performance carefully and provide timely and necessary support, using available resources. Moreover, the risk factors that we examined in this study are general demographic factors that medical schools typically collect during the admission process. Thus, without using special research tools [10], teachers could use these variables in the early years of medical schools to identify students who may need support later, and prepare necessary and timely support.

Regarding research implications, previous studies have reported that demographic factors (age, sex) as well as school factors have been linked to students’ academic performance [3–12]. Our study adds to the literature by clarifying the associations between these factors and students’ academic performance trajectories instead of discrete academic performance measured at some time points. Future studies by multiple institutions are warranted to confirm our findings.

Finally, studies investigating mechanisms of the association between these factors and the students’ academic performance are crucial. Possible mechanisms include isolation, students’ mental health, diet, sleep patterns, and socioeconomic status for high school geographical area, and mental health and sleep problems for high school graduation year. Clarifying the mechanisms would provide useful information for preparing and designing effective support to medical students who need it.

## Conclusions

In conclusion, using group-based trajectory modeling, we found that high school geographical area, type of admission test, high school graduation year, and sex were associated with GPA trajectories. These findings will provide important insights to identify and monitor students at risk for poor academic performance to provide adequate and timely support.

## Supporting information

S1 Fig The GPA trajectories of medical students that were modeled using GPA data from 3rd semester to 7th semester. (TIF)

S1 Table The odds ratios of being a member of certain group of GPA trajectory relative to a reference group by demographic factors in medical students (N = 202) without adjustment for high school GPA (ref: The highest GPA trajectory group N = 39 (19.3%)). (The GPA trajectories of medical students were modeled using GPA data from 3rd semester to 8th semester).
(DOCX)

S2 Table The odds ratios of being a member of certain group of GPA trajectory relative to a reference group by demographic factors in medical students (N = 202) with adjustment for high school GPA (ref: The highest GPA trajectory group N = 45 (22.3%)). (The GPA trajectories of medical students were modeled using GPA data from 3rd semester to 7th semester).
(DOCX)

S3 Table The odds ratios of being a member of certain group of GPA trajectory relative to a reference group by demographic factors in medical students (N = 202) without adjustment for high school GPA (ref: The highest GPA trajectory group N = 45 (22.3%)). (The GPA trajectories of medical students were modeled using GPA data from 3rd semester to 7th semester).
(DOCX)

## References

[1] Association of Japanese Medical colleges (AJMC). Survey on academic perfoemance of medical studetns. <https://www.ajmc.jp/pdf/180305_2.pdf> [accessed April 10, 2020] [in Japanese]. 2018.

[2] Tsunekawa K, Suzuki Y. Practice and Challenge of Institutional Research in Gifu University School of Medicine Annual Report, Organization for Promotion of Higher Education and Student Support, Gifu University, Gifu, Japan 2016.

[3] Ferguson E, James D, Madeley L. Factors associated with success in medical school: systematic review of the literature. BMJ. 2002;324(7343):952–7. 10.1136/bmj.324.7343.952 11964342 PMC102330

[4] Malau-Aduli BS, O’Connor T, Ray RA, van der Kruk Y, Bellingan M, Teague PA. Risk factors associated with academic difficulty in an Australian regionally located medical school. BMC Med Educ. 2017;17(1):266 10.1186/s12909-017-1095-9 29282058 PMC5745748

[5] Arulampalam W, Naylor R, Smith J. Factors affecting the probability of first year medical student dropout in the UK: a logistic analysis for the intake cohorts of 1980–92. Med Educ. 2004;38(5):492–503. 10.1046/j.1365-2929.2004.01815.x 15107083

[6] Arulampalam W, Naylor RA, Smith JP. Dropping out of medical school in the UK: explaining the changes over ten years. Med Educ. 2007;41(4):385–94. 10.1111/j.1365-2929.2007.02710.x 17430284

[7] Yates J, James D. Risk factors for poor performance on the undergraduate medical course: cohort study at Nottingham University. Med Educ. 2007;41(1):65–73. 10.1111/j.1365-2929.2006.02648.x 17209894

[8] Lumb AB, Vail A. Comparison of academic, application form and social factors in predicting early performance on the medical course. Med Educ. 2004;38(9):1002–5. 10.1111/j.1365-2929.2004.01912.x 15327683

[9] James D, Chilvers C. Academic and non-academic predictors of success on the Nottingham undergraduate medical course 1970–1995. Med Educ. 2001;35(11):1056–64. 10.1046/j.1365-2923.2001.01042.x 11703642

[10] Cleland JA, Milne A, Sinclair H, Lee AJ. Cohort study on predicting grades: is performance on early MBChB assessments predictive of later undergraduate grades? Med Educ. 2008;42(7):676–83. 10.1111/j.1365-2923.2008.03037.x 18588648

[11] Kumwenda B, Cleland JA, Walker K, Lee AJ, Greatrix R. The relationship between school type and academic performance at medical school: a national, multi-cohort study. BMJ Open. 2017;7(8):e016291 10.1136/bmjopen-2017-016291 28860227 PMC5589012

[12] Ray RA, Woolley T, Sen Gupta T. James Cook University’s rurally orientated medical school selection process: quality graduates and positive workforce outcomes. Rural Remote Health. 2015;15(4):3424 26442581

[13] van Rooij EC, Jansen EP, van de Grift WJEJoPoE, 33(4), 749–767. First-year university students’ academic success: the importance of academic adjustment. European Journal of Psychology of Education. 2018;33(4):749–67.

[14] Rienties B, Beausaert S, Grohnert T, Niemantsverdriet S, Kommers P. Understanding academic performance of international students: the role of ethnicity, academic and social integration. Higher Education. 2012;63(6):685–700.

[15] Petersen IH, Louw J, Dumont K. Adjustment to university and academic performance among disadvantaged students in South Africa. Educational Psychology. 2009;29(1):99–115.

[16] Baker RW, Siryk B. Measuring adjustment to college. Journal of Counseling Psychology. 1984;31(2):179.

[17] Baker SR. Intrinsic, extrinsic, and amotivational orientations: Their role in university adjustment, stress, well-being, and subsequent academic performance. Current Psychology. 2004;23(3):189–202.

[18] Nagin DS. Analyzing developmental trajectories: a semiparametric, group-based approach. Psychological Methods. 1999;4(2):139–57. 10.1037/1082-989x.6.1.18 11285809

[19] Nagin DS, Tremblay RE. Analyzing developmental trajectories of distinct but related behaviors: a group-based method. Psychological Methods. 2001;6(1):18–34. 10.1037/1082-989x.6.1.18 11285809

[20] Jones BL, Nagin DS. A note on a Stata plugin for estimating group-based trajectory models. Sociological Methods & Research. 2013;42(4):608–13.

[21] Nagin DS, Odgers CL. Group-based trajectory modeling in clinical research. Annual Review of Clinical Psychology. 2010;6:109–38. 10.1146/annurev.clinpsy.121208.131413 20192788

[22] Griffin B, Bayl-Smith P, Hu W. Predicting patterns of change and stability in student performance across a medical degree. Medical Education. 2018;52(4):438–46. 10.1111/medu.13508 29349791

[23] Sinclair HK, Cleland JA. Undergraduate medical students: who seeks formative feedback? Med Educ. 2007;41(6):580–2. 10.1111/j.1365-2923.2007.02768.x 17518838

[24] Martin IG, Jolly B. Predictive validity and estimated cut score of an objective structured clinical examination (OSCE) used as an assessment of clinical skills at the end of the first clinical year. Med Educ. 2002;36(5):418–25. 10.1046/j.1365-2923.2002.01207.x 12028391

[25] Kuramoto N, Koizumi R. Current issues in large-scale educational assessment in Japan: Focus on national assessment of academic ability and university entrance examinations. Assessment in education: principles, policy & practice. 2018;25(4):415–33.

[26] Guest M. Japanese university entrance examinations: What teachers should know. Language Teacher. 2008;32(2):15.

[27] Nawa N, Numasawa M, Nakagawa M, Sunaga M, Fujiwara T, Tanaka Y, et al Differential effects of individual and school factors on the academic trajectories of Japanese dental students. J Dent Educ. 2020. 10.1002/jdd.12150 32216137

[28] Sutcliffe AG, Gardiner J, Melhuish E. Educational progress of looked-after children in England: a study using group trajectory analysis. Pediatrics. 2017;140(3). 10.1542/peds.2017-0503 28778858

[29] Maslov Kruzicevic S, Barisic KJ, Banozic A, Esteban CD, Sapunar D, Puljak L. Predictors of attrition and academic success of medical students: a 30-year retrospective study. PLoS One. 2012;7(6):e39144 10.1371/journal.pone.0039144 22737228 PMC3377595

[30] Al Shawwa L, Abulaban AA, Abulaban AA, Merdad A, Baghlaf S, Algethami A, et al Factors potentially influencing academic performance among medical students. Advances in Medical Education and Practice. 2015;6:65–75. 10.2147/AMEP.S69304 25674033 PMC4321417

[31] Salem RO, Al-Mously N, Nabil NM, Al-Zalabani AH, Al-Dhawi AF, Al-Hamdan N. Academic and socio-demographic factors influencing students’ performance in a new Saudi medical school. Medical Teacher. 2013;35 Suppl 1:S83–9. 23581903 10.3109/0142159X.2013.765551

[32] Considine G, Zappalà G. The influence of social and economic disadvantage in the academic performance of school students in Australia. Journal of Sociology. 2002;38(2):129–48.

[33] James R. Participation disadvantage in Australian higher education: An analysis of some effects of geographical location and socioeconomic status. Higher Education. 2001;42(4):455–72.

[34] Hayes LD. Higher education in Japan. The Social Science Journal. 1997;34(3):297–310.

[35] Koyama A, Matsushita M, Ushijima H, Jono T, Ikeda M. Association between depression, examination-related stressors, and sense of coherence: the Ronin-Sei study. Psychiatry and Clinical Neurosciences. 2014;68(6):441–7. 10.1111/pcn.12146 24506541

[36] Matsushita M, Koyama A, Ushijima H, Mikami A, Katsumata Y, Kikuchi Y, et al Sleep duration and its association with sleepiness and depression in "ronin-sei" preparatory school students. Asian Journal of Psychiatry. 2014;9:61–6. 10.1016/j.ajp.2014.01.006 24813039

