# Supplementary figures and images for: Associations between demographic factors and the academic trajectories of medical students in Japan
Source: PLoS One. 2020 May 18;15(5):e0233371. doi: 10.1371/journal.pone.0233371 (PMC7233530; doi:10.1371/journal.pone.0233371)

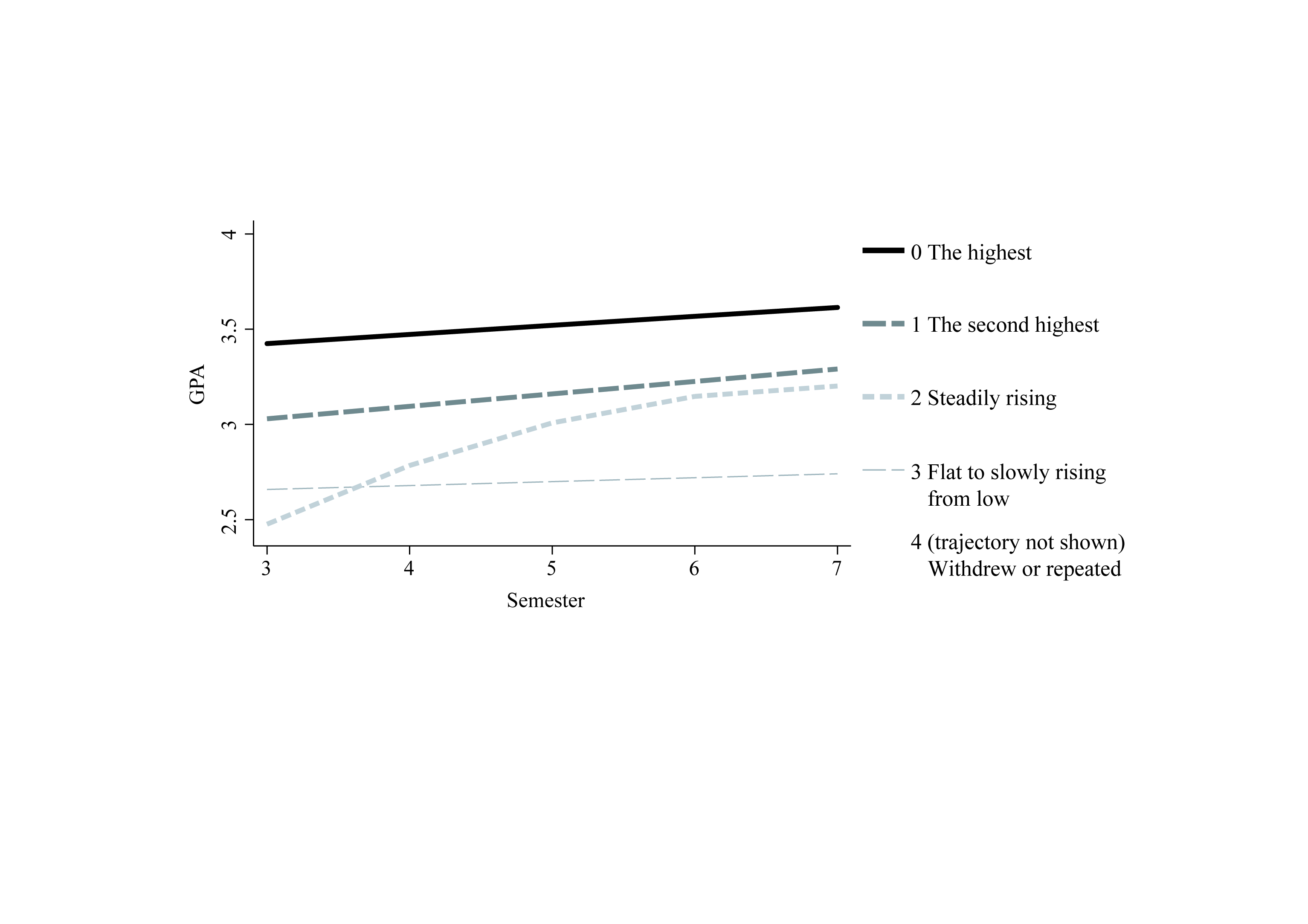

Supplement: S1 Fig — (TIF) [file pone.0233371.s001.tif]
