# Supplementary material for: Associations between demographic factors and the academic trajectories of medical students in Japan
Source: PLoS One. 2020 May 18;15(5):e0233371. doi: 10.1371/journal.pone.0233371 (PMC7233530; doi:10.1371/journal.pone.0233371)
Supplement: S1 Table — (The GPA trajectories of medical students were modeled using GPA data from 3rd semester to 8th semester). (DOCX) [file pone.0233371.s002.docx]

**S1 Table. The odds ratios of being a member of certain group of GPA trajectory relative to a reference group by demographic factors in medical students (N=202) without adjustment for high school GPA (ref: the highest GPA trajectory group N=39 (19.3%)). (The GPA trajectories of medical students were modeled using GPA data from 3rd semester to 8th semester.)**

|  | Group 1:  The second highest  (N=67 (33.2%)) |  | Group 2:  Steadily rising  (N=42 (20.8%)) |  | Group 3:  Flat to slowly rising from low  (N=33 (16.3%)) |  | Group 4:  Withdrew or repeated  (N=21 (10.4%)) |
| --- | --- | --- | --- | --- | --- | --- | --- |
| **Variable** | OR  (95% CI) |  | OR  (95% CI) |  | OR  (95% CI) |  | OR  (95% CI) |
| Type of  high school  (ref: Public) |  |  |  |  |  |  |  |
| Private | 1.12  (0.42, 3.02) |  | 1.52  (0.46, 4.99) |  | 1.50  (0.43, 5.24) |  | 0.88  (0.22, 3.54) |
| National | 0.34  (0.06, 1.82) |  | 1.10  (0.20, 6.09) |  | 1.27  (0.23, 7.15) |  | 2.04  (0.36, 11.51) |
| Geographical  area of  high school  (ref: Inside the  National Capital  Region) |  |  |  |  |  |  |  |
| Outside the region | 1.34  (0.43, 4.18) |  | 0.91  (0.24, 3.46) |  | 2.96  (0.89, 9.80) |  | **5.08**  **(1.41, 18.24)** |
| Type of  admission test  (ref:  First exam) |  |  |  |  |  |  |  |
| Second exam | **0.28**  **(0.10, 0.81)** |  | 0.35  (0.11, 1.16) |  | 0.55  (0.18, 1.70) |  | **0.12**  **(0.01, 0.99)** |
| Quota for  certain  prefectures | 0.22  (0.02, 2.51) |  | 1.69  (0.28, 10.33) |  | 0.49  (0.04, 5.80) |  | ND^a^ |
| High school graduation year (ref: Recent graduates) |  |  |  |  |  |  |  |
| Past graduates | 2.15  (0.77, 5.98) |  | **5.37**  **(1.81, 15.94)** |  | **7.57**  **(2.47, 23.22)** |  | 2.99  (0.84, 10.65) |
| Biology major  (ref: No) |  |  |  |  |  |  |  |
| Yes | 0.55  (0.21, 1.47) |  | 0.65  (0.22, 1.95) |  | 0.51  (0.15, 1.68) |  | 1.23  (0.37, 4.07) |
| Sex  (ref: Female) |  |  |  |  |  |  |  |
| Male | 1.07  (0.47, 2.46) |  | 1.76  (0.66, 4.68) |  | 2.09  (0.72, 6.03) |  | 3.34  (0.83, 13.36) |
| One point  decline  in high school  GPA | 4.68  (1.00, 21.91) |  | **9.23**  **(1.85, 46.12)** |  | **27.52**  **(5.37, 141.05)** |  | **21.12**  **(3.72, 119.99)** |

Adjusted for year of admission.

Bolded values indicate statistical significance at p<0.05.
