# Supplementary material for: Associations between demographic factors and the academic trajectories of medical students in Japan
Source: PLoS One. 2020 May 18;15(5):e0233371. doi: 10.1371/journal.pone.0233371 (PMC7233530; doi:10.1371/journal.pone.0233371)
Supplement: S2 Table — (The GPA trajectories of medical students were modeled using GPA data from 3rd semester to 7th semester). (DOCX) [file pone.0233371.s003.docx]

**S2 Table. The odds ratios of being a member of certain group of GPA trajectory relative to a reference group by demographic factors in medical students (N=202) with adjustment for high school GPA (ref: the highest GPA trajectory group N=45 (22.3%)). (The GPA trajectories of medical students were modeled using GPA data from 3rd semester to 7th semester.)**

|  | Group 1:  The second highest  (N=78 (38.6%)) |  | Group 2:  Steadily rising  (N=38 (18.8%)) |  | Group 3:  Flat to slowly rising from low  (N=20 (9.9%)) |  | Group 4:  Withdrew or repeated  (N=21 (10.4%)) |
| --- | --- | --- | --- | --- | --- | --- | --- |
| **Variable** | OR  (95% CI) |  | OR  (95% CI) |  | OR  (95% CI) |  | OR  (95% CI) |
| Type of  high school  (ref: Public) |  |  |  |  |  |  |  |
| Private | 1.25  (0.48, 3.23) |  | 1.63  (0.48, 5.59) |  | 3.77  (0.67, 21.35) |  | 1.12  (0.27, 4.62) |
| National | 0.34  (0.07, 1.71) |  | 0.84  (0.14, 5.14) |  | 1.06  (0.10, 11.29) |  | 1.37  (0.22, 8.72) |
| Geographical  area of  high school  (ref: Inside the  National Capital  Region) |  |  |  |  |  |  |  |
| Outside the region | 1.92  (0.63, 5.79) |  | 1.92  (0.51, 7.14) |  | **4.37**  **(1.09, 17.54)** |  | **8.16**  **(2.14, 31.11)** |
| Type of  admission test  (ref: First exam) |  |  |  |  |  |  |  |
| Second exam | 0.38  (0.14, 1.01) |  | 0.42  (0.11, 1.55) |  | 0.77  (0.20, 2.96) |  | 0.15  (0.02, 1.33) |
| Quota for  certain  prefectures | 0.50  (0.07, 3.78) |  | 3.12  (0.49, 19.86) |  | NA |  | NA |
| High school graduation year (ref: Recent graduates) |  |  |  |  |  |  |  |
| Past graduates | **2.61**  **(1.01, 6.72)** |  | **4.91**  **(1.66, 14.53)** |  | **3.66**  **(1.04, 12.87)** |  | 2.06  (0.57, 7.46) |
| Biology major  (ref: No) |  |  |  |  |  |  |  |
| Yes | 0.98  (0.39, 2.42) |  | 0.53  (0.15, 1.96) |  | 0.72  (0.17, 3.12) |  | 1.81  (0.52, 6.25) |
| Sex  (ref: Female) |  |  |  |  |  |  |  |
| Male | 0.95  (0.42, 2.13) |  | 2.32  (0.79, 6.82) |  | 2.60  (0.69, 9.81) |  | 3.84  (0.91, 16.11) |

Adjusted for year of admission and high school GPA.

Bolded values indicate statistical significance at p<0.05.
