# Supplementary material for: Associations between demographic factors and the academic trajectories of medical students in Japan
Source: PLoS One. 2020 May 18;15(5):e0233371. doi: 10.1371/journal.pone.0233371 (PMC7233530; doi:10.1371/journal.pone.0233371)
Supplement: S3 Table — (The GPA trajectories of medical students were modeled using GPA data from 3rd semester to 7th semester). (DOCX) [file pone.0233371.s004.docx]

**S3 Table. The odds ratios of being a member of certain group of GPA trajectory relative to a reference group by demographic factors in medical students (N=202) without adjustment for high school GPA (ref: the highest GPA trajectory group N=45 (22.3%)). (The GPA trajectories of medical students were modeled using GPA data from 3rd semester to 7th semester.)**

|  | Group 1:  The second highest  (N=78 (38.6%)) |  | Group 2:  Steadily rising  (N=38 (18.8%)) |  | Group 3:  Flat to slowly rising from low  (N=20 (9.9%)) |  | Group 4:  Withdrew or repeated  (N=21 (10.4%)) |
| --- | --- | --- | --- | --- | --- | --- | --- |
| **Variable** | OR  (95% CI) |  | OR  (95% CI) |  | OR  (95% CI) |  | OR  (95% CI) |
| Type of  high school  (ref: Public) |  |  |  |  |  |  |  |
| Private | 1.05  (0.41, 2.65) |  | 1.18  (0.37, 3.81) |  | 2.43  (0.46, 12.65) |  | 0.82  (0.21, 3.20) |
| National | 0.45  (0.10, 2.12) |  | 1.56  (0.30, 8.00) |  | 1.77  (0.19, 16.78) |  | 2.30  (0.41, 12.81) |
| Geographical  area of  high school  (ref: Inside the  National Capital  Region) |  |  |  |  |  |  |  |
| Outside the region | 1.75  (0.59, 5.23) |  | 1.50  (0.41, 5.44) |  | 3.43  (0.90, 13.03) |  | **6.00**  **(1.68, 21.44)** |
| Type of  admission test  (ref: First exam) |  |  |  |  |  |  |  |
| Second exam | **0.35**  **(0.13, 0.91)** |  | 0.33  (0.10, 1.17) |  | 0.65  (0.18, 2.36) |  | 0.13  (0.02, 1.06) |
| Quota for  certain  prefectures | 0.47  (0.06, 3.46) |  | 2.23  (0.36, 13.65) |  | NA |  | NA |
| High school graduation year (ref: Recent graduates) |  |  |  |  |  |  |  |
| Past  graduates | **2.95**  **(1.15, 7.55)** |  | **6.48**  **(2.25, 18.66)** |  | **5.28**  **(1.59, 17.54)** |  | 2.98  (0.87, 10.18) |
| Biology major  (ref: No) |  |  |  |  |  |  |  |
| Yes | 0.91  (0.37, 2.22) |  | 0.45  (0.13, 1.61) |  | 0.59  (0.14, 2.45) |  | 1.48  (0.45, 4.85) |
| Sex (ref: Female) |  |  |  |  |  |  |  |
| Male | 0.81  (0.37, 1.76) |  | 1.66  (0.60, 4.59) |  | 1.82  (0.51, 6.45) |  | 2.68  (0.68, 10.65) |
| One point  decline  in high school  GPA | **4.94**  **(1.19, 20.49)** |  | **17.68**  **(3.84, 81.50)** |  | **21.24**  **(3.94, 114.58)** |  | **20.41**  **(3.88, 107.27)** |

Adjusted for year of admission.

Bolded values indicate statistical significance at p<0.05.
